# Assessment of Exposure to Di-(2-ethylhexyl) Phthalate (DEHP) Metabolites and Bisphenol A (BPA) and Its Importance for the Prevention of Cardiometabolic Diseases

**DOI:** 10.3390/metabo12020167

**Published:** 2022-02-10

**Authors:** Fabrizia Carli, Demetrio Ciociaro, Amalia Gastaldelli

**Affiliations:** Institute of Clinical Physiology, National Research Council, Via Giuseppe Moruzzi 1, 56124 Pisa, Italy; fcarli@ifc.cnr.it (F.C.); ciociaro@ifc.cnr.it (D.C.)

**Keywords:** endocrine disrupting chemicals (EDCs), biomonitoring, exposomics, ultra high-pressure liquid chromatography quadrupole time-of-flight spectrometry (UHPLC/QTOF), gas chromatography mass spectrometry (GC/MS), di-(2-ethylhexyl) phthalate (DEHP) metabolites, mono(2-ethylhexyl) phthalate (MEHP), mono(2-ethyl-5-hydroxyhexyl) phthalate (MEHHP), mono(2-ethyl-5-oxohexyl) phthalate (MEOHP), bisphenol A (BPA), urine

## Abstract

Exposomics analyses have highlighted the importance of biomonitoring of human exposure to pollutants, even non-persistent, for the prevention of non-communicable diseases such as obesity, diabetes, non-alcoholic fatty liver disease, atherosclerosis, and cardiovascular diseases. Phthalates and bisphenol A (BPA) are endocrine disrupting chemicals (EDCs) widely used in industry and in a large range of daily life products that increase the risk of endocrine and cardiometabolic diseases especially if the exposure starts during childhood. Thus, biomonitoring of exposure to these compounds is important not only in adulthood but also in childhood. This was the goal of the LIFE-PERSUADED project that measured the exposure to phthalates (DEHP metabolites, MEHP, MEHHP, MEOHP) and BPA in Italian mother–children couples of different ages. In this paper we describe the method that was set up for the LIFE PERSUADED project and validated during the proficiency test (ICI/EQUAS) showing that accurate determination of urinary phthalates and BPA can be achieved starting from small sample size (0.5 mL) using two MS techniques applied in cascade on the same deconjugated matrix.

## 1. Introduction

**Figure 1:**
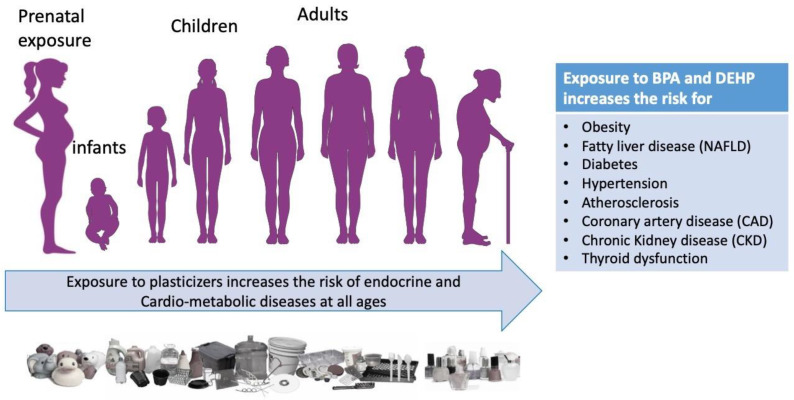
Long term exposure to phthalates and BPA increases the risk of cardiometabolic diseases.

Plasticizers are colorless and odorless esters, mainly phthalates or bisphenols, that increase the elasticity of a material (e.g., polyvinylchloride (PVC). For these properties they are widely used in industry and in a large range of daily life products. The majority of these are considered as non-persistent pollutants and have a short half-life. However, the exposure to these agents has significant consequences on human health since, once ingested or inhaled, they are harmful for health as they act as endocrine disrupting chemicals (EDCs), i.e., they are able to interfere with the endocrine system by modulating and/or disrupting the metabolic and hormonal functions responsible for the maintenance of homeostasis, reproduction, development, and/or behavior [1]. As many studies have shown, they increase the risk to develop endocrine and cardiometabolic diseases such as obesity [2,3], non-alcoholic fatty liver disease (NAFLD) [4,5,6,7] diabetes, and impairment in insulin secretion and beta cell function [8,9,10], hypertension [11,12,13,14], atherosclerosis [9,15,16], coronary artery disease (CAD) [17,18,19,20], chronic kidney disease (CKD) [21,22,23], and thyroid dysfunction [24,25,26] (Figure 1). The longer the exposure (e.g., prenatal or during childhood) the higher the risk [27].

### 1.1. Fate of Phthalates and Bisphenols in Humans

Phthalates are derived from phthalic acid and are used primarily to make plastic products more flexible. For the past 50 years, phthalate production has increased, and plastic products may contain up to 40–50% of phthalate by weight. Phthalates are present in all consumer products containing plastics including packaging materials for food, children’s and baby’s toys, household items, waterworks, and paints but also in medical devices, such as tubing and intravenous bags, and in personal product care such as cosmetics and perfumes [28,29]. Phthalates are bound non-covalently to the polymer matrix making them highly susceptible to leaching so they can easily migrate from plastic to air and food, but also to blood and skin. That explains why human exposure to phthalates and bisphenols is so widespread and nearly ubiquitous, and why more than 75% of the U.S. population had phthalate metabolite detected in urines (more recent references) [30,31]. 

Once absorbed into the human body phthalates are rapidly metabolized with half-lives within few hours [31,32]. Their metabolism depends on the structure formula, i.e., the shorter-chain-length phthalates undergo preferentially to a phase-I metabolism in which they are hydrolyzed to the corresponding monoesters, than conjugated with glucuronic acid in a phase II metabolism and excreted into urine and feces; the longer-chain-length phthalates after being first hydrolyzed and then oxidized. 

**Figure 2:**
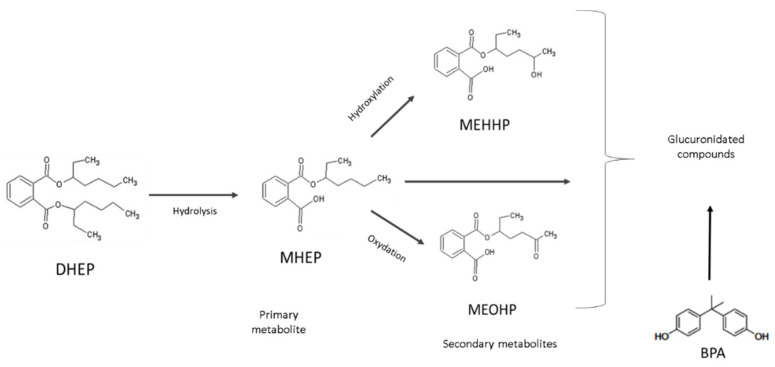
Metabolism of DEHP and BPA. DEHP is hydrolyzed in MEHP and is again hydrolyzed in MEHHP or is oxidated in MEOHP; the three metabolites are conjugated with glucuronic acid. BPA is directly conjugated with glucuronic acid.

After absorption DEHP is rapidly metabolized by hydrolysis to monoester, mono(2-ethylhexyl) phthalate (MEHP) [33] that is further metabolized to mono(2-ethyl-5-hydroxyhexyl) phthalate (MEHHP) and mono(2-ethyl-5-oxohexyl) phthalate (MEOHP) (Figure 2). A second step is hepatic glucuronic conjugation that is important to reduce their potential biological activity as well as to facilitate their excretion from the human body increasing their water solubility [31,34,35,36]. The great majority of DEHP metabolites is found in urine in glucuronidated form. 

Another group of plasticizers are bisphenols that include Bisphenol A (BPA), and the new Bisphenol S and F. Bisphenols contain two hydroxyphenyl functionalities, most of them based on diphenylmethane (Figure 2). BPA is a monomeric building block of polycarbonate and is widely used as an additive to plastics (for example polyvinyl chloride) and other consumer products, such as thermal paper [32]. Thus food and drink packaging is an important source of BPA that can migrate to the food because some monomers are unbound and factors such as elevated temperature favors leaching process [37]. After absorption, BPA is mostly glucuronidated in liver and then excreted in urines (Figure 2). Thus, total (i.e., free plus glucuronidated) urinary BPA and phthalate metabolites MEHP, MEHHP, and MEOHP are considered the biomarkers for assessing human exposure to BPA and to parent phthalate DEHP.

### 1.2. Disrupting Action of DEHP and BPA

Phthalates and bisphenols are among the most studied endocrine disrupting chemicals (EDCs). This disruption can happen through the alteration of normal hormone levels, halting or stimulating the production of hormones, or changing the way hormones travel through the body, thus affecting hormones control. Moreover, they can modify the individual epigenetic characteristics through DNA methylation, histone modification, and microRNAs causing alteration in gene expression with adverse health effects [38]. 

Several studies suggest that phthalates and BPA contribute to the increase in obesity [3,39], development of insulin resistance and type 2 diabetes [40,41,42], and renal dysfunction as our recent results found a positive association between exposure to DEHP and degree of albuminuria in subjects with type 2 diabetes [43]. 

Phthalates and DEHP cause toxicity in many organs: for example, in the liver they alter enzyme activities or gene expression and induce oxidative stress [44,45], hepatomegaly, and hepatocarcinogenesis [46]; in the brain PPARγ hyperactivation leads to apoptosis in undifferentiated neurons [47]. Thus, tolerable daily intake (TDI) for DEHP has been set to 50 μg per kilogram body weight per day (EFSA 2005) while EPA has set the reference dose (RfD) to 20 μg per kilogram body weight per day (mg/kg/d) based on increased relative liver weights in guinea pigs [48].

Phthalates can bind and activate several nuclear receptors, particularly in the liver and adipose tissue, but also in other organs such as the thyroid and in the reproductive system [49,50]. Phthalates, as many EDCs, act as antagonists or agonists of nuclear hormone receptors (NRs). Phthalates are selective modulators of peroxisome proliferator-activated receptors (PPARs), a family of nuclear receptors involved in glucose and lipid metabolism. In humans three PPAR subtypes have been identified: PPARα and PPARβ/δ mainly involved in the fatty acid catabolism in muscle and liver; and PPARγ involved mainly in lipid metabolism and adipogenesis [51,52]. The monoesters of phthalates, such as the MEHP derived from DEHP, have also been indicated as activators of PPARγ [53,54,55]. The phthalate monoester MEHP binds PPARγ similar to Rosiglitazone, an antidiabetic drug in the thiazolidinedione (TZD) class, but this binding induces the recruitment of only a subset of coregulators activating a subset of target genes with adipogenic effect [52,53]. Recent studies have shown that DEHP is able to interfere with the insulin signal suggesting DEHP exposure impairs insulin signal transduction and alters glucoregulatory events leading to the development of type 2 diabetes in F1 male offspring [56,57]. 

BPA is also able to bind to PPARs. Activation of PPARs by BPA induces insulin resistance in all organs, glucose intolerance, hepatic hypertriglyceridemia, adipogenesis, and excess release of non-esterified fatty acids, similarly to high fat diet [58]. BPA exposure can also induce epigenetic modifications associated to type 2 diabetes and obesity. Tolerable daily intake (TDI) for BPA was established from European Food Safety Authority at 4 μg/kg bw per day (EFSA 2015) but recently proposed to reduce to 0.04 ng/kg bw per day due to the emerging toxicity on the immune system (EFSA 2021).

In Sprague–Dawley rats BPA induced the downregulation of glucokinase (gck) gene in liver leading to glucose intolerance and insulin resistance [59,60]. BPA was shown to induce epigenetic modifications in genes involved in lipid metabolism reducing the mRNA expression of adipose tissue Srebpc1, PPARα, and Cpt1β and the hepatic expression of CD36 [58]. Oral exposure of BPA in mice leads to upregulation in genes involved in lipid synthesis, that increase de novo lipogenesis and promote NAFLD-like phenotype [61]. DEHP and BPA are also able to activate estrogen receptor ERα and Erβ that are involved in growth and development but also in numerous physiological functions [62,63]. 

### 1.3. Biomonitoring Studies

The biomonitoring studies aim at the measurement of the exposure to pollutants in the general population by measuring environmental chemicals, or their metabolites, in biological samples. Environmental chemicals are normally present in the matrix at trace levels, so the first step is to define which biological matrix is the most appropriate, e.g., urine, blood, or saliva.Urines are more easily collected than blood, e.g., for children, and are preferred for non-persistent environment exposure as BPA and phthalates since their concentration is more abundant in urines where they are excreted as both free and glucuronidated compounds compared to other matrices [64]. Exposure is assessed by the measurement of both free and glucuronic compounds. Typically, exposure is measured in a single spot urine sample (first void urine), but to reduce variability it would be more appropriate to perform a 24 h urine collection or pool of different samples [65]. The analysis of presence of environmental chemicals is usually done by mass spectrometry, either liquid chromatography mass spectrometry (LC/MS) or gas chromatography mass spectrometry. The concentration of the environmental chemicals is usually done by comparing the peak area of the chemical with the peak area of the corresponding labeled internal standard added at known concentration to the matrix. When spot urines are used for the analysis, the chemical concentrations are normalized to the urinary concentration of creatinine. The preparation and purification of the sample and the choice chromatographic approach have an impact on the quality of the measurement.

### 1.4. Research Gaps and Aim

Di-(2-ethylhexyl) phthalate (DEHP) and bisphenol A (BPA) are EDCs widely used in industry and in a large range of daily life products that increase the risk of endocrine and cardiometabolic diseases. The biomonitoring of exposure to DEHP and BPA is important not only in adulthood but also in childhood. Several methods have been described for the measurement of DEHP metabolites (MEHP, MEHHP, MEOHP) and BPA in urine samples, although the stability, reproducibility, and precision of analyses over time was not reported previously. 

Before starting the analyses of the LIFE-PERSUADED project we evaluated the methods previously published for the purification and quantification of phthalates and BPA in biological or non-biological matrix. Urinary BPA and DEHP metabolites are usually quantified by mass spectrometry, couple with either gas or liquid chromatography. Most of the methods used LC/MS with triple quadrupole for DEHP metabolites [66,67,68,69,70,71,72], but none at that time used a QTOF, while both LC/MS and GC/MS were used for BPA. High resolution LC/QTOF mass spectrometry is the best method for discovery of known and unknown compounds in the matrix of interest; given its selectivity and accuracy it is becoming a useful tool to study the exposure to EDCs even in small biological samples [73]. Unlike LC/MS/MS system with target MRM acquisitions, the LC/QTOF system allows you to acquire and quantify several compounds at high resolution in a single run compatibly with the chromatographic method. 

Urine samples are easy to collect in large amounts in adults, but this is not always feasible in young children. Since the LIFE-PERSUADED project enrolled children for whom (4–6 years old) it was sometime difficult to get a large sample size, we developed a method that allowed to measure DEHP metabolites, BPA, and creatinine concentrations starting from small sample size (0.5 mL for each analysis) using two MS techniques applied in cascade on the same deconjugated matrix. Stability, reproducibility, and precision of analyses over time are reported in this paper as well as the results of the validation during the Interlaboratory Comparison Investigations and External Quality Assurance Schemes (ICI/EQUAS) of the HBM4EU Project [74].

## 2. Experimental Design

### 2.1. The LIFE-PERSUADED Project

The LIFE-PERSUADED project aimed at the biomonitoring of phthalates (DEHP metabolites) and BPA in Italian healthy non-obese children (age 4–14 years) and their mothers (900 couples) collected from 2015 to 2018 during the human biomonitoring study [75,76,77]. Moreover, we studied the DEHP and BPA exposure in children with idiopathic premature thelarche (IPT) or precocious puberty (IPP, a minimum of 30 girls in each group, aged 2–7 years) and idiopathic obesity (IO, a minimum of 30 boys and 30 girls, aged 6–10 years). Moreover, the project included a toxicological study in juvenile rats exposed to DEHP and BPA at the same doses evaluated in the children of the project, to evaluate the effects of exposure in the animal model.

Here we report the details and reliability of the method developed for the measurement of DEHP metabolites and BPA in urine samples starting from 0.5 mL. More than 3000 samples were processed throughout the 4-year project including urine samples, standards, and quality control showing good stability and reproducibility of the measurement over time, i.e., quality control samples run together with samples. 

### 2.2. Chemicals for Preparation of Standard Solutions

Solutions and standard curves were prepared using unlabeled standards, i.e., Mono(2-ethylhexyl) phthalate (MEHP, ULM-4583-MT-1.2), Mono-2ethyl-5hydroxyhexyl phthalate (MEHHP, ULM-4662-MT-1.2), and mono-2ethy-5oxo hexyl phthalate (MEOHP, ULM-4663-MT-1.2) (Cambridge Isotope Laboratories, Tewksbury, MA, USA); we used a nominal concentration of 100 ng/mL for each compound. As internal standards we used ^13^C-DEHP metabolites and prepared a mix solution containing 100 ng/mL of ^13^C_4_-MEHP (ring-1,2-^13^C_2_, dicarboxyl-^13^C_2_), 100 ng/mL ^13^C_4_-MEHHP, and 100 ng/mL ^13^C_4_-MEOHP (Cambridge Isotope Laboratories, Tewksbury, MA, USA). The standard solution of BPA (purchased from Dr. Ehrenstofer Reference Materials Residue Analysis, Augsburg, Germany) was prepared by weighing and dissolving the compound in acetonitrile; the solutions concentration obtained was 535 ng/mL. BPAd_16_ (C_15_ ^2^H_16_ O_2_, CDN isotopes, Pointe-Claire, Quebec CDN) was used as Internal Standard and was prepared by weighing and dissolving the compound in acetonitrile at final concentration of 216 ng/mL. The internal standards (IS) (40 µL of ^13^C DEHP metabolites and 20 µL of BPAd_16_, per sample) were added to the urine samples for quantification of compound concentrations.

### 2.3. Preparation of Quality Control Samples

For each metabolite we prepared and run Quality Control (Matrix QC) samples prepared by spiking the biological matrix (that was urine from a standard pool) with a known amount of MEHP, MEOHP, and MEHHP to reach a final concentration of 15.2 ng/mL for each metabolite and with a BPA to reach 21.4 ng/mL. QC samples were prepared in batches before starting the analyses of the samples of the LIFE PERSUADED project and 200 µL aliquots were stored at −20 °C for subsequent analyses. Repeated injections of matrix QC were made to evaluate method robustness and to assess stability of samples stored at −20 °C over time.

### 2.4. Collection of Urine Samples

During the project, urine samples were collected into polypropylene (PP) tubes (i.e., without phthalates and BPA), and stored locally at −20 °C until transferred to the CNR for analyses (around 8–12 weeks). Samples were then aliquoted and stored at −20 °C until mass spectrometry analysis and additional aliquots were stored at −80 °C in a biobank for the long storage.

### 2.5. Equipment

For phthalates and BPA extraction we used Agilent Vac Elut Manifold with SPE cartridge C18 ODS 3 mL tubes 200 mg (Agilent, Santa Clara, CA, USA).

UHPLC/ESI-QTOF: the LC system is an Ultra-High Pressure (Agilent UHPLC 1290 infinity) with a binary pump, autosampler and Thermostatted Column Compartment (TCC) that mounted an Agilent ZORBAX SB-Phenyl column (2.1 × 100 mm, 1.8-Micron, Agilent, Santa Clara, CA, USA). The Quadrupole Time-of-Flight (QTOF, Agilent 6540, Santa Clara CA) is equipped with a Dual ESI Jet Stream; the electrospray ionization was performed in negative mode. Instrument Mode was Extended Dynamic Range (2 GHz) in Mass Range Low (1700 *m*/*z*).

GC/MS single quadrupole (GC 7890-MS 5975, Agilent, Santa Clara, CA, USA) with autosampler. The chromatography was performed by a capillary column (DB-5MS J&W, l 30 m; i.d. 0.25 mm; film thickness 0.25 um, Agilent, Santa Clara, CA, USA). Samples were then ionized by electron impact (EI) at 70 eV and chromatograms were acquired in selected ion monitoring (SIM).

## 3. Procedure

### 3.1. Preparation of Standard Curves

Standard solutions of MEHP, MEHHP, and MEOHP were prepared in acetonitrile. For each metabolite standard curves were prepared at 5 points with concentrations of 4.1, 6.1, 8.1, 16.2, and 50.7 ng/mL of unlabeled compounds, that ideally covered the possible quantities present in the urine, and with 8 ng/mL of each internal standards. The final solution was dried under a gentle nitrogen flux and resuspended with 150 µL acetonitrile:water (1:9, *v*/*v*) as urine samples. Calibration curves were injected once a week before starting the analyses of urine samples and used to check linearity of the instrument in the defined concentrations and to correct the results for recovery. The standard curve of BPA was prepared using 7 different concentrations of BPA (from 1.1 to 71.5 ng/mL) and 20 µL of BPAd_16_ (216 ng/mL) and was injected in GC/MS once a week before start sequences analysis. 

### 3.2. Preparation of Samples for Mass Spectrometry Analysis

Samples and QCs were thawed at 4 °C and 500 μL of urine was transferred in a glass tube. To each sample we added 375 μL of ammonium acetate buffer (NH4CH3COO-) 1M (Merck, Darmstadt, Germany), at PH = 5, 250 μL of MilliQ water (Millipore Milli-Q Synthesis A10, Merck, Darmstadt, Germany), and 2 μL of the enzyme β-glucuronidase (from Helix from Pomatia enzyme aqueous solution, ≥100,000 units/mL; Merck, Darmstadt, Germany) to deconjugate DEHP metabolites and BPA. The samples were incubated at 37 °C overnight (for at least 16 h). We also used Abalonase Purified β-Glucuronidase (United Chemical Technologies, Bristol, PA, USA) >50,000 units/mL with 10× Rapid Hydrolysis Buffer (1:1, *v*/*v* with urine) and urine samples with mix labeled standard solution were incubated at 60 °C for 1 h. After overnight incubation, 25 μL of formic acid was added to stop the enzyme reaction and then 40 μL of the mix labeled standard solution was added to the samples. Finally, 1 mL of MilliQ water was added into each sample to facilitate passage into solid phase extraction. For phthalates and BPA extraction we used Agilent Vac Elut Manifold with SPE cartridge C18 ODS 3 mL tubes 200 mg (Agilent, Santa Clara, CA, USA). SPE cartridges were conditioned with 2 mL of methanol and then 2 mL of MilliQ water. Urine samples were then loaded into the cartridges and washed with 1.5 mL of MilliQ water. Elution was performed with 1 mL of acetonitrile followed by 1 mL of methanol. The samples were then dried under a gentle N2 flux, reconstituted with 150 μL of acetonitrile:water (1:9, *v*/*v*) and transferred into glass vials for the analyses by UHPLC/QTOF [78]. We setup a GC/MS method that used the same de-glucuronidated urines previously prepared to measure DEHP metabolite concentrations by UHPLC/QTOF. Briefly, the samples were transferred to a glass tube and dried under a nitrogen flow. Then, 10 µL of BSTFA 1%TMS and 50 µL of acetonitrile (Merck, Darmstadt, Germany) were added to derivatize the samples and incubated at 75 °C for 40 min. The sample was then transferred into glass vials avoiding contact with other materials for GC/MS analysis.

### 3.3. Instrumental Conditions

#### 3.3.1. Analyses of DEHP metabolites by LC/QTOF

LC conditions: the LC system mounted an Agilent ZORBAX SB-Phenyl column (2.1 × 100 mm, 1.8-Micron, Agilent, Santa Clara, CA, USA) maintained at 20 °C with an in-line Filter (0.3 μm SS Frit, 1.3 μL Delay Volume). The needle was washed with 100% methanol for 20 s and the injection volume was 5 μL. The mobile phase A was 10 mM ammonium acetate and the mobile phase B was methanol at a flow rate of 0.3 mL/min. The total run time was 14 min (including 2 min of equilibration time). The mobile phase gradient is reported in Table 1. 

**Table 1:** Mobile phase gradient of the method: A = Water 10 mM Ammonia Acetate; B = Methanol.

Time	Solvent A (%)	Solvent B (%)
0	67	33
4	67	33
6	40	60
7	5	95
10	67	33
12	67	33

MS conditions: The ESI-QTOF configuration and conditions are reported in detail in Table 2; the electrospray ionization was performed in negative mode. The acquisition mass range was *m/z* 100–500. Retention times were 7.6 min for MEHP, 4.4 min for MEOHP, and 4.7 min for MEHHP.

**Table 2:** QTOF parameters.

Parameters	Values
Ionization mode	Negative ionization
Capillary voltage	−2250
Nozzle voltage	−300
Nebulizer pressure	35
Dry gas temperature	300 °C
Dry gas flow rate	8 L/min
Sheath gas temperature	350 °C
Sheath gas flow rate	11 L/min

#### 3.3.2. Analyses of BPA by LC/QTOF

Quantification of BPA in urines was first performed by UHPLC/QTOF simultaneously with phthalates using the conditions reported in Table 1 and Table 2. Although free BPA could be accurately quantified by LC/MS, for total BPA (i.e., after deconjugation) the analyses showed a matrix effect (described in the result session) that affected the reproducibility and accuracy of the measurements. Retention time for BPA was 6.8 min.

#### 3.3.3. Analyses of BPA by GC/MS

To assess total BPA, we used a GC/MS single quadrupole (GC 7890-MS 5975, Agilent, Santa Clara, CA, USA). The front SS Inlet was set in splitless mode at 280 °C, and carrier gas was helium at a constant flow rate of 2 mL/min. The oven program was 80 °C for 1 min, then 30 °C/min to 250 °C for 0 min and 40 °C/min to 300 °C for 2 min. Oven equilibration time was 0.5 min and run time was 9.9 min. Compounds were separated using a capillary column (DB-5MS J&W, length 30 m; i.d. 0.25 mm; film thickness 0.25 um). Samples were ionized by electron impact (EI) at 70 eV and chromatograms were acquired in selected ion monitoring (SIM). MS source was set up at 230 °C and MS quad at 150 °C and temperature in the transfer line was 280 °C. The fragment ion for BPA was 357 *m*/*z* and for d_16_BPA was 368 *m*/*z*. Retention time for BPA was 6.8 min. and 6.78 for d_16_BPA in GC/MS.

### 3.4. Assessment of Limit of Detection (LOD) and Quantification (LOQ) of the Method

Limits of Detection and Quantification (respectively LOD and LOQ) were established following the IUPAC guidelines ([79] also recommended by the U.S. Environment Protection Agency EPA (https://www.epa.gov/cwa-methods/procedures-detection-and-quantitation-documents, accessed on 20 December 2021, Office of Pesticide Programs U.S. Environmental Protection Agency Washington, DC 20460, USA). 

To calculate LOD and LOQ of phthalates we used the standard deviation of the first point of the curve that had a signal-to-noise ratio SNR >5 given by Agilent Mass Hunter Qualitative Analysis B.06.00 that calculates the distance from the height of the peak to the midline between the maximum and minimum noise at baseline. LOD and LOQ were set to 3 and 10 standard deviations above the response at 0 concentration (blank) estimated by the standard curve. For each metabolite (MEHP, MEHHP, MEOHP) we prepared 4 samples in acetonitrile:water (1:9, *v*/*v*) blank, concentration 0.1, 1, 3, and 5 ng/mL with a constant plus 8 ng/mL of ^13^C labeled standards. We also determined the Method Detection Limit (MDL), i.e., the minimum concentration of a substance that can be measured with a 99% confidence that the analyte concentration is greater than zero; MDL was calculated by multiplying the standard deviation of lowest measurable sample by the Student’s *t*-value at the 99 percent confidence level.

LOD and LOQ for total BPA were calculated according to U.S.EPA procedure (U.S. EPA 2017). Briefly we assessed blank concentration of BPA during GC/MS runs by measuring standard deviation of more than 10 runs. 

### 3.5. Assessment of Reagent Blank and Spike Recovery

Reagent Blank and spike recovery were assessed by measuring DEHP metabolites and BPA in water and urine samples where known amount of labeled and unlabeled MEHP, MEOHP, MEHHP, and BPA were added. Urines were extracted in SPE cartridge C18 (see preparation of urine samples). Recovery of DEHP metabolites was calculated as the ratio between the spike concentrations and nominal values (3.8 ng/mL). For LC/MS analysis matrix effect (ME) was calculated from ratio of concentration in spike urine sample and spike in water. For BPA analysis, before starting the sequence, the blanks with the internal standard (d_16_BPA) are run for about 10 injections to settle the BPA contamination signal. Recovery of BPA was calculated as the ratio between concentration of spikes in urine and nominal values (10.4 ng/mL).

### 3.6. Calculations of EDCs Concentrations and Assessment of Exposure

Concentrations of DEHP metabolites and BPA were assessed with mass spectrometry and were normalized using creatine urine concentration to adjust the urinary concentrations of EDCs for the dilution. Results in LIFE PERSUADED project were reported un-adjust (µg/L) and adjusted (µg/g crea) to creatinine concentrations measured using the Jaffe method (Beckman Coulter, Brea, CA, USA) [75,76,78,80]. Assessment of DEHP exposure were estimated using sum of concentrations of its metabolites.

Relative metabolic rates (RMR) of the transformations from precursors to products (Figure 2) of MEHP, MEOHP, and MEHHP were calculated as RMR1 = ([MEHHP] + [MEOHP])/[MEHP] and RMR2 = ([MEOHP]/[MEHHP]) × 10, representing the rate of hydroxylation from MEHP to MEHHP and hydroxylation from MEHP to MEHHP respectively.

**Figure 3:**
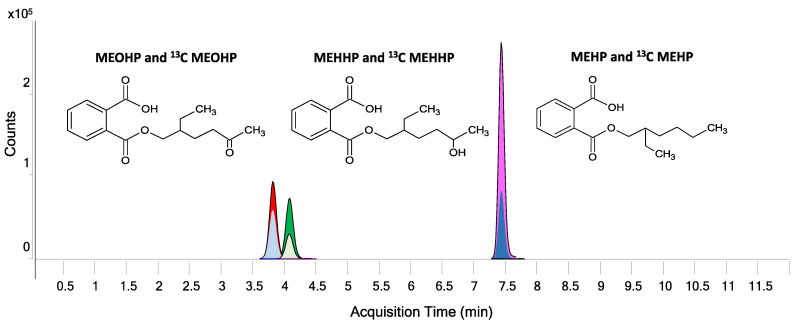
Peak extraction of standards of phthalate metabolites (MEOHP, MEHHP, and MEHP) and their ^13^C labeled standard by UHPLC/QTOF.

LC/MS chromatograms were analyzed with the instrumental software Mass Hunter Profinder B.06.00 (Agilent Technology, Santa Clara, CA, USA) with targeted feature extraction (Figure A3) Mass Profinder algorithm was used to identify and extract compounds by known chemical formulas [M-H]^−^ ± 0.01 *m*/*z* tolerance since ionization was performed in negative mode (Figure 3), to calculate the relative isotope composition of compounds and peak area of each compound (Table A1). GC/MS chromatograms were analyzed by the GC/MSD ChemStation software (Agilent Technology, Santa Clara, CA, USA).

The concentration of each compound was calculated from the peak area ratio of the labeled internal standards to the unlabeled compounds as:

Cx = CIS × VIS/Vx × (Ax/AIS)

where Cx = compound concentration; CIS = internal standard concentration; VIS = volume of IS; Vx = volume of compound x; Ax = compound area; AIS = Area of the internal standard compound.

## 4. Results

### 4.1. Quantification of DEHP Metabolites by UHPLC/QTOF

We report the results of the method for the quantification of DEHP metabolites in urines using a high resolution UHPLC/QTOF equipped with a phenyl column (details in the method section). Figure 3 shows the good separation in the chromatogram of unlabeled and labeled standards for MEOHP, MEHHP, and MEHP.

#### 4.1.1. Recovery and Matrix Effect of DEHP Metabolites 

The recovery of MEHP, MEOHP, and MEHHP ranged between 79–102% for both unlabeled and labeled standards (Table 3). Background levels in blank, measured by adding only internal labeled standards, was 0.28 ng/mL for MEHP and no detectable (ND) for the other metabolites. Matrix effect (ME%) for DEHP metabolites in LC/MS analysis was 90% for MEHP, 102% for MEHOP, and 105% for MEHHP.

#### 4.1.2. Limit of Detection (LOD) and Quantification (LOQ) of DEHP Metabolites 

Limit of Detection (LOD) and Quantification (LOQ) were determined for MEHP, MEHHP, and MEOHP. LOD for DEHP metabolites ranged between 0.11 to 0.28 ng/mL and LOQ from 0.24 to 0.58 ng/mL (Table 4). Method Detection Limit (MDL) was the lowest for MEOHP (0.096 ng/mL) and the highest for MEHP (0.238 ng/mL).

#### 4.1.3. Working Range and Linearity of DEHP Method

**Figure A1:**
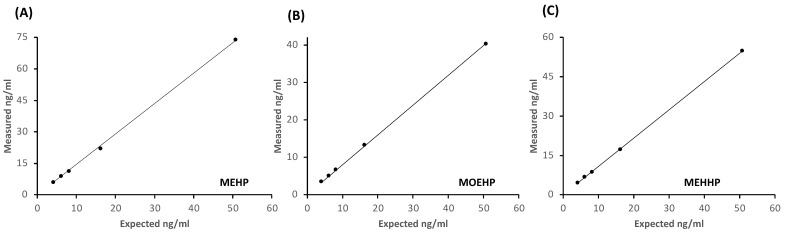
Standard curves of DEHP metabolites measured by UHPLC/MS-QTOF: (**A**) MEHP (**B**) MEOHP (**C**) MEHHP. Measured concentrations expressed as ng/mL (N = 29) of DEHP metabolites are reported in the y axis while expected (theoretical) values are shown in the x axis; R = 0.99 for all metabolites.

For each compound (either MEHP, MEOHP, or MEHHP) we tested the linearity and stability of the instrumental measurement by running labeled standard curves once per week before starting the analyses. Each curve contained five points where the ^13^C labeled internal standard was at the same concentration used in urine samples while the unlabeled standard was added at increasing concentrations (4.1, 6.1, 8.1, 16.2, and 50.7 ng/mL). The standard curves showed good linearity (r > 0.995 for MEHP, MEOHP, and MEHHP) over the range 4–50 ng/mL (Figure A1).

#### 4.1.4. Stability, Reproducibility of the Analysis, and Precision of DEHP Method

**Figure 4:**
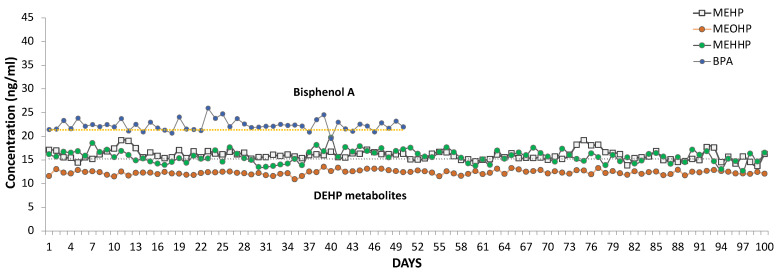
Reproducibility of the matrix QC spiked with MEHP, MEHHP and MEOHP and measured by UHPLC/QTOF over three years, i.e., from 2015 to 2018 (N = 100). The average values ± standard deviation (ng/mL) measured for quality control (QC) samples of standard metabolites were: MEHP: 15.96 ± 1.04; MEHHP: 15.73 ± 1.31; MEOHP: 12.34 ± 0.45, respectively. For BPA we assessed stability and reproducibility in n = 50 QC together with DEHP metabolites in LC/MS analysis and average value was 22.30 ± 1.17 ng/mL and RSD = 5.25%. The low variation of QC samples, prepared at the beginning of the study and stored at −20 °C, showed that DEHP metabolites and BPA are stable also if stored at −20 °C.

Several aliquots of quality control (QC) samples were prepared from a urine pool as describe in the methods and analyzed during every run. Figure 4 reports the concentrations of MEHP, MEOHP, and MEHHP in QC samples measured for the LIFE-PERSUADED analyses from 2015 to 2018. Concentrations of DEHP metabolites in QC samples showed stability and reproducibility over time as shown by the 3-years plot in Figure 4. MEHHP variability was the highest, though less than 10%. The Relative Standard Deviation (RSD) of DEHP metabolites in QC samples was 6.49% for MEHP, 3.69% for MEOHP, and 8.21% for MEHHP.

### 4.2. Quantification of Bisphenol A (BPA) by UHPLC/QTOF and GC/MS

**Figure 5:**
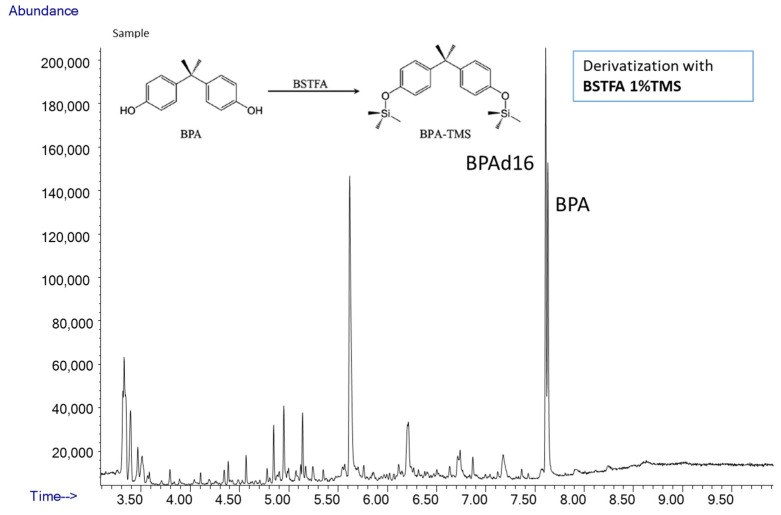
GC/MS TIC Chromatogram of total BPA in a deconjugated urine sample. BPA and BPAd_16_ were derivatized by TMS and the peak with 2 TMS groups was detected and quantified using fragment 357 *m*/*z* for BPA and 368 *m*/*z* for BPAd_16_ in SIM mode. Molecular ions were detected with fragment target ions for identification (372 *m*/*z* for BPA and 386 *m*/*z* for BPAd_16_).

Total BPA in urine samples was quantified first using by high resolution UHPLC/QTOF, i.e., during the same run of DEHP metabolites, and then by the GC/MS using the method approved by HBM4EU Project (https://www.hbm4eu.eu/online-library/?mdocs-cat=mdocs-cat-20&mdocs-att=null#, last accessed on 1 December 2021) (Figure 5).

#### 4.2.1. Working Range and Linearity of BPA Methods

**Figure A2:**
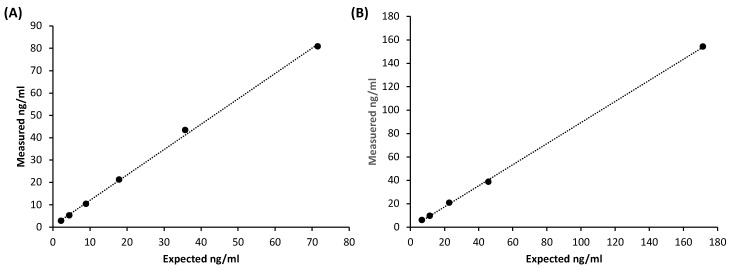
Standard curves of BPA measured by GC/MS (**A**) and UHPLC/MS-QTOF (**B**), as ng/mL. The graphs show measured concentrations (*y* axis) vs expected values (*x* axis); R = 0.99 for both analyses.

**Figure A3:**
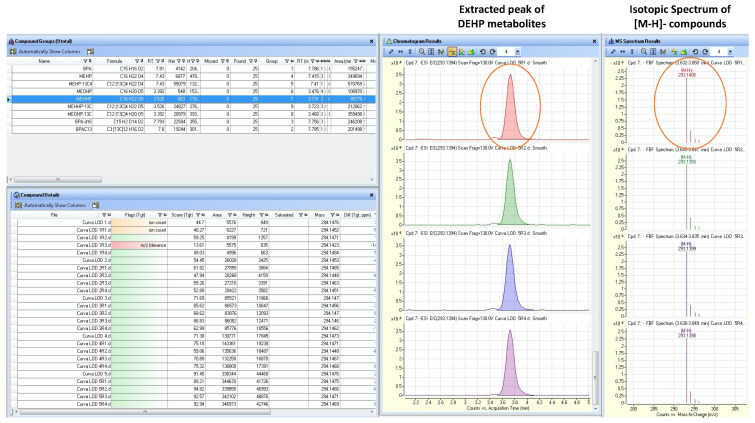
Chromatograms were analyzed with instrumental software Mass Hunter Profinder B.06.00 Profinder (Agilent, Santa Clara, CA, USA) with targeted feature extraction. Mass Profinder algorithm was used to identify and extract compounds by known chemical formulas ([M-H]^−^ ± 0.001 *m/z* tolerance.

The linearity of both LC/MS and GC/MS methods was tested by preparing a standard curve covering the range from 1.1 to 71.53 ng. The standard curves showed good linearity in both instruments, r = 0.99 (Figure A2). 

#### 4.2.2. Stability, Reproducibility of the Analysis, and Precision of BPA Methods

Quantification of QC samples for BPA showed good reproducibility, recovery, and accuracy by both LC/MS and GC/MS analyses. QCs were measured regularly during GC/MS run and showed good reproducibility and accuracy. QCs (N = 100) had a mean of 24 ± 4.3 ng/mL with RSD% of 17.96%. Recovery of BPAd_16_, added in the same amount in free and deconjugated urine, was much lower in deconjugated urine than in free urine; furthermore, the addition of increasing concentrations of IS after deconjugation did not result in a proportional increase in the signal which, on the contrary, remained very low, indicating strong signal suppression. Moreover, the LC/MS analysis of the deconjugated samples showed that the peak area of BPAd_16_ was variable and low: (a) despite being added in constant concentrations, and (b) compared to the IS areas of the DEHP metabolites that also had been added to the same matrix in constant concentrations and analyzed under the same instrumental conditions. Since derivatization of the same samples (deconjugated and non-deconjugated) with GC/MS analysis did not detect the same problems, we concluded that it is likely that compounds created during deconjugation affect sample recovery and ionization. 

#### 4.2.3. Limit of Detection (LOD) and Quantification (LOQ) of BPA by GC/MS

We calculated LOD and LOQ according to U.S.EPA procedure (U.S. EPA 2017) and were 0.157 ng/mL and 0.523 ng/mL, respectively. For BPA contamination we analyzed a sample of BPAd_16_ to quantify the blank signal. To stabilize the background signal of the BPA we made 20 successive injections of the internal standard until reaching the minimum blank signal corresponding to 0.69 ng/ml.

### 4.3. Validation of the Methods during the HBM4EU Project

The analytical methods for DEHP metabolites and BPA were tested and validated during an Inter-laboratory Comparison Investigation (ICI) and External Quality Assurance Scheme (EQUAS) organized within the framework of the HBM4EU project (https://www.hbm4eu.eu/online-library/?mdocs-cat=mdocs-cat-20&mdocs-att=null#, accessed on 20 December 2021). Laboratories from 16 countries were invited to participate to the blind analyses of urine samples for phthalates and bisphenols. Table 5 reports the results for the analyses conducted in our laboratory for low and high concentration of DEHP metabolites and bisphenol A in urine samples. 

## 5. Discussion

The European Commission (EFSA) and the US Environmental Protection Agency (EPA) [81,82,83,84,85] have warned and restricted the use of Di(2-ethylhexyl) phthalate (DEHP) in food packaging and personal care products, but they are still widely present in many consumer products [29,86]. Moreover, in December 2019 EPA designated DEHP as a High-Priority Substance and the chemical is currently undergoing risk evaluation. The creation of materials suitable for the needs of consumer products led to the synthesis of new chemicals that unfortunately are dangerous for human health since they interfere with the hormonal system [87]. In 1997 the Environmental Protection Agency (EPA) defined the term endocrine disrupting chemicals (EDC) as “an exogenous agent that interferes with the production, release, transport, metabolism, binding, action, or elimination of natural hormones in the body responsible for the maintenance of homeostasis and the regulation of developmental processes” (EPA 1997). Since then, more and more reports showed how these EDCs act in organisms and the dangers they entail for human health. Great attention has been given to exposure to these substances in growing children where EDCs can act easily and lead to developmental abnormalities. The biomonitoring of these substances allows to understand the exposure to a given pollutant and lead to a regulation of these substances to limit damage to health and increase national health expenditure. Despite restrictions in the employment of DEHP and BPA and replacement by other plasticizers (e.g., dioctyl terephthalate, DEHT, bisphenol S and F), DEHP and BPA are still widely present in packaging and personal care products and they can also easily pass to food, as shown in a study that analyzed food samples taken from fast food restaurants finding DEHP and BPA in more than 70% samples [86]. Although mass spectrometry is the preferred method for the detection of environmental pollutants, the type of chromatographic method used may vary (i.e., gas or liquid chromatography). Moreover, there are several types of mass spectrometers, for LC/MS triple quadrupole LC/MS/MS that acquires data in multiple reaction monitoring (MRM), but also hybrid triple-quadrupole linear ion trap mass spectrometer (QTRAP) in selected reaction monitoring (SRM) mode [88], providing a good sensitive response. Several studies used GC/MS, with single quadrupole or tandem mass spectrometer, that requires derivatization of the sample, but recently also eliminating the derivatization step [89,90,91]. At the time of the start of the LIFE PERSUADED project there was no published method using high resolution LC/QTOF. High resolution mass spectrometry is the best method for discovery of known and unknown compounds in the matrix of interest; given its selectivity and accuracy it is becoming a useful tool to study the exposure to EDCs even in small biological sample [73]. Unlike an LC/MS/MS system with target MRM acquisitions, the LC/QTOF system allows to acquire and quantify several compounds with high resolution in a single analysis compatibly with the chromatographic method. The method developed for the European project LIFE-PERSUADED project and here reported used UHPLC/QTOF for the evaluation of DEHP metabolites and GC/MS for BPA in urine samples. For both methods there was good agreement with published studies. The methods were tested and were validated during the proficiency test (ICI/EQUAS), organized within the activities of the HBM4EU project, which provided urine samples with reference values from certified laboratories. The initial setup of the method included the simultaneous measurement of DEHP metabolites and BPA by UHPLC/QTOF, as also recently proposed [92]. We found that the method gave reliable responses for free BPA, but we detected significant signal suppression due to matrix effects after deconjugation for the measurement of total environmental chemicals (i.e., free plus glucuronidated compounds). The analyses were then repeated by GC/MS where the matrix effect was absent. Since the LIFE-PERSUADED study (“Phthalates and bisphenol A biomonitoring in Italian mother–child pairs: link between exposure and juvenile diseases”) involved children, there was a need to start from small urine volumes. Although urine samples are easy to collect in adults, in children this is not always so simple, especially when they are very young; thus, the use of a very sensitive instrument allows the detection of these compounds starting from low volumes. Thus, the method was setup in samples as low as 0.5 mL. Our method has the advantage to be easy to perform and shows high stability and reproducibility of the quality control samples measured over 3 years. 

The method has also been tested and validated during the proficiency test in Interlaboratory Comparison Investigations and External Quality Assurance Schemes (ICI/EQUAS) of the HBM4EU Project showing good agreement with reference values. The quality of the measurement of environmental chemicals in biological matrices is determinant for the biomonitoring studies but often challenging. Measurements of phthalates or bisphenols concentrations in biological matrices are not currently standardized. In 2018 HBM4EU organized an Inter-Laboratory Comparison Investigation (ICI) to compare data and methods within several laboratories (18 for phthalates and 24 for bisphenols) in different European states and harmonize measurements to make comparable data generated by different laboratories. HMB4EU will use these biomonitoring techniques to assess human exposure to chemicals in Europe and better understand health effects and improve chemical risk assessment. For the analysis of the metabolites of DEHP in the urine, there is no commercially available certified material to test an analytical method. Participation to the HBM4EU ICI/EQUAS tests allowed us to evaluate and validate analyses in urine samples, because provided a reference value from certified laboratories. The method here developed showed good sensitivity with LOQ ranging from 0.24 to 0.58 ng/mL, in line with methods previously published [66,67,69,70]. For MEHP, the lowest value provided was below the LOQ, but the values obtained by our experimental method were very close to the reference values, in low and high concentrations. BIAS% was good ranging from 5.14 to 12.96 in the analyses conducted with different sample preparations, different times, and different chromatographic runs, therefore including all possible variabilities. For BPA, on the other hand, we found numerous problems in LC/MS relating to the matrix effect in the analysis of urine samples; for this reason, we have developed a method that allows us to analyze BPA in GC/MS that allows good recovery and sensitivity. The LC/MS matrix effect affected the recovery of both analytes and internal standards and we observed a signal suppression likely due to the presence of molecules derived from enzymatic deconjugation that reduced the accuracy of the measurement. Indeed, the problem was evident in urine only after enzymatic deconjugation since the addition of different spikes of standard BPA did not result in a dose dependent recovery, while in free urine this matrix effect was not present. We showed that the matrix effect disappeared when the matrix analyzed by UHPLC/QTOF was derivatized and analyzed by GC/MS. 

The results of the LIFE PERSUADED project showed that the majority of Italian children population (4–14 years old) and women were exposed to DEHP and BPA [76,77], with measurable levels of its representative metabolites and showing a continuous and widespread exposure for DEHP (47.49 μg/L or 45.32 μg/g crea, as geometric mean, GM). Indeed, for DEHP metabolites both UHPLC/MS and GC/MS methods have been reported, although analysis by LC/MS is the most common, while for BPA both instruments have been used although the accredited method (ISO17025) is by GC/MS/MS [93]. The HBM4EU proficiency test suggested to follow the method by GC/MS/MS validated in the LABERCA laboratory (France’s National Reference Laboratory for Food Residues and Contaminants) while no suggestions were given for phthalates. For both phthalates and BPA, we obtained good results within the acceptable ranges required by the ICI/EQUAS tests (Table 5). 

In conclusion, the new method here presented has the advantage to start from low volumes of urine (0.5 mL) and, by using LC/QTOF followed by GC/MS, allows the accurate measurement of both DEHP metabolites and BPA.

## Figures and Tables

**Table 3:** Recovery of DEHP metabolites and BPA in blank and urine samples.

	MEHP	MEOHP	MEHHP	BPA
Spike (ng/mL)	3.8	3.8	3.8	10.4
Blank (ng/mL)	0.28 ± 0.01	ND	ND	0.69 ± 0.006
Urine + spike (ng/mL)	4.16 ± 0.15	3.75 ± 0.20	4.60 ± 0.13	9.6 ± 0.17
Recovery %	94%	79%	102%	91.99%
Matrix Effect %	90%	102%	105%	NA

**Table 4:** Limit of Detection (LOD), Quantification (LOQ), and Method Detection Limit (MDL); SNR: signal-to-noise ratio.

DHEP Metabolites	LOD (ng/mL)	LOQ (ng/mL)	MDL (ng/mL)	SNR
MEHP	0.28	0.58	0.238	11.30
MEOHP	0.18	0.48	0.096	14.50
MEHHP	0.11	0.24	0.131	10.60

**Table 5:** Quantification of DEHP metabolites and BPA in 2 reference samples with low and high concentration of metabolites; replicates were measured for DEHP metabolites and 8 for BPA and the average was reported. RDS%= relative standard deviation; LOQ= limit of quantification.

	Concentration	Ref Value ng/mL	Replicates	Average	Precision (RSD%)	Trueness (Bias%)
				ng/mL		
MEHP	Low	0.567	9	<LOQ	-	-
	High	5.89	9	6.65	14.43	12.96
MEOHP	Low	1.74	9	1.63	23.91	6.22
	High	14.6	9	13.32	7.55	8.74
MEHHP	Low	4.12	9	3.71	14.96	9.87
	High	32.3	9	30.64	13.01	5.14
BPA	Low	0.763	8	0.751	19.17	1.55
	High	8.4	8	6.456	13.29	23.14

## Data Availability

No new data were created or analyzed in this study. Data sharing is not applicable to this article.

## Appendix A

**Table A1:** Chemical features (molecular formulas, molecular weight (MW) and mass to charge ratio (*m*/*z*)) of MEHP, MEHOP, MEHHP and of their labelled standard compounds.

Compound	Molecular Formula	MW	*m*/*z* [M-H]^−^
MEHP	C_16_H_22_O_4_	278	277.1445
^13^C_4_MEHP	C_12_[^13^C]_4_H_22_O_4_	282	281.1580
MEOHP	C_16_H_20_O_5_	292	291.1238
^13^C_4_ MEOHP	C_12_[^13^C]_4_H_20_O_5_	296	295.1372
MEHHP	C_16_H_22_O_5_	294	293.1394
^13^C_4_ MEHHP	C_12_[^13^C]_4_H_22_O_5_	298	297.1529
